# Predicting common bean (*Phaseolus vulgaris*) productivity according to *Rhizoctonia* root and stem rot and weed development at field plot scale

**DOI:** 10.3389/fpls.2022.1038538

**Published:** 2022-11-30

**Authors:** Bita Naseri, Seyed Hossein Nazer Kakhki

**Affiliations:** ^1^ Plant Protection Research Department, Kermanshah Agricultural and Natural Resources Research and Education Center, AREEO, Kermanshah, Iran; ^2^ Plant Protection Research Department, Zanjan Agricultural and Natural Resources Research and Education Center, AREEO, Zanjan, Iran

**Keywords:** crop management, legumes, root canker, dry bean, yield

## Abstract

**Introduction:**

A two-year research trial was conducted to evaluate progression of common bean (Phaseolus vulgaris) growth, Rhizoctonia root rot and weed development in association with productivity when treated with different cultivars, planting dates and weed control methods in north-western part of Iran.

**Methods:**

To determine the best descriptors, six standard curves were examined to model development of bean dry matter, Rhizoctonia root rot incidence, and weed density during two growing seasons across 256 field plots. Exponential and linear-by-linear models were fitted to bean-disease-weed progression data, and then model parameters representing over-season progress curve elements were used in multivariate regression analyses to estimate bean production.

**Results and discussion:**

Furthermore, using herbicides (Imazethapyr and Trifluralin) restricted weed density by 28% in early (mid-spring) and 42% in late (late spring to early summer) plantings. Late plantings of two bean cultivars decreased disease progress up to 36% for herbicide use, hand-weeding and control. Although bean dry matter, pod and seed production for herbicide use and hand-weeding treatments were 6-17% greater than control, late planting improved productivity in control by 10-24%. Findings suggested that late planting of bean improved efficiency of herbicides to control weeds. Late planting also restricted Rhizoctonia root rot progress and thus, improved bean yield. There were significant correlations between bean-disease-weed development descriptors. According to principal component analysis, bean-disease-productivity-weed variables accounted for 80% of total data variance. Such information extends our understanding of bean-disease-weed progress in interaction with planting date to develop more effective and sustainable integrated Rhizoctonia management programs.

## Introduction

Knowing *Rhizoctonia* root rot and weed development as damaging agents in common bean (*Phaseolus vulgaris* L.) cultivation worldwide (Naseri, 2019b; Oveisi et al., 2021), an improved understanding of bean production affected by the agrosystem-disease-weed interaction leads to develop more influential integrated crop management programs. A large number of *Rhizoctonia* root rot and weed control strategies in the forms of agronomic practices (Naseri and Veisi, 2019), bean resistance, biological and chemical management methods (Dehghani et al., 2018; Tabande and Naseri, 2020) have been reported previously. However, the estimation of bean production according to the progression of *Rhizoctonia* root rot in conjunction with the development of bean and weeds across experimental plots differing in bean cultivars, sowing dates and weed management treatments is still lacking.

*Rhizoctonia* root rot caused by *Rhizoctonia solani* has been known as a destructive disease in bean growing lands around the world as reviewed by Naseri and Younesi (2021). A number of agro-ecological factors restricted *Rhizoctonia* root rot spread and thus, improved productivity in commercial bean cropping systems surveyed by Naseri and Moradi (2015) as follows: manure application, sprinkler irrigation, growing beans following potato and tomato, the lack of urea application, proper planting density, shallow sowing, avoiding furrow irrigation, manual cultivation, planting Red beans, adequate soil organic matter, irrigating at 6–9 days intervals, increasing rhizobial nodulation, and growing beans in soils with 15–30% silt content. Further to management of bean root rot, it is essential to optimize the integrated management of diseases in conjunction with weeds for sustainable bean production. Chemical weed control is widely used by Iranian bean farmers. Although herbicide use can effectively restrict crop losses to weeds, they increase bean farming expenses, weed resistance to herbicide, environmental pollutants and human health risks (Oveisi et al., 2021). Therefore, it is highly desired to minimize herbicides usage with the assistance of effective agricultural practices for sustainable bean production. For instance, bean competitiveness and herbicide efficiency improved following the application of herbicides when combined with the mixed-cropping (Oveisi et al., 2021). A large number of reports on agricultural, biological and chemical control of either *Rhizoctonia* root rot or weeds in bean farming systems are available (Stagnari and Pisante, 2011; Esmaeilzadeh and Aminpanah, 2015; Byiringiro et al., 2017). However, more reliable yield estimates according to the disease and weed development under influential sustainable crop management programs consisting a well-timed planting deserves much more attention. Therefore, an advanced understanding of the best descriptors of *Rhizoctonia* root rot progress in conjunction with weed development under different planting date and weed control treatments at field plot scale is still needed to predict crop damage more accurately. Previous plot- and large-scale (Naseri, 2013a; Naseri, 2013b) findings in Iran indicated that later (early summer) sowing of bean crops restricted *Rhizoctonia* root rot and weed development (Kakhki et al., 2022), and improved seed production. To extend such benefits of later bean sowings, an experimental plot scale study was conducted to examine the interrelationships among descriptors of bean dry matter, pod and seed production, *Rhizoctonia* root rot and weed density development in two commercial cultivars (COS16 and Talash) planted under various sowing date and weed control treatments during two growing seasons.

## Materials and methods

### Experimental design

In 2015 and 2016 years, bean dry matter, *Rhizoctonia* root rot, and weed density were examined across experimental plots naturally infested with *R. solani* and weed populations, confirmed by the pre-test of soil (clay-loamy, mixed, mesic, Typic Haploxerepts) samples collected from the experimental plots. The experiments were performed at Kheirabad Research Site (latitude 36˚31´ N, longitude 48˚47´ E; 1,770 m a.s.l; 284.5 mm annual rainfall, 142 annual frost days). The experiment was a split-split-plot designed as follows: planting date as the main plots which consisted of four dates of 10-15 May, 26-31 May, 10-15 June, and 25-30 June, and weed-control practices as the subplots: Imazethapyr application, Trifluralin application, hand-weeding and control, and two commercial common bean cultivars as the sub-subplots: Talash (climbing bean) and COS16 (bush bean). The two chemical weed control treatments involved the pre-emergence use of Imazethapyr (Pursuit™ 10% SL) at 1 l ha^-1^, and pre-planting Trifluralin (Treflan™ 48% EC) at 2.5 l ha^-1^. In this two-year research, each experimental plot was replicated four times. Each experimental plot was consisted of six 5-m-length rows spaced by 0.30 m with plant spacing of 0.075 m resulting in an approximate density of 40 plant/m^2^. To avoid herbicide-mixing, plots were spaced by 5 m.

For each plot, weed density was determined as the number of weeds in a 0.6 × 0.6 m quadrat (three quadrats per plot) at seedling (with one and three leaflets or cotyledon leaves opened), 50% flowering, podding and physiological maturity stages of bean plants per plot. To detect *Rhizoctonia* root rot incidence, five bean plants per quadrat were dug up randomly to assess red-brown cankers on the root (Naseri, 2013b). The percentage of plants having *Rhizoctonia* root cankers in five examined plants per quadrat (the same quadrats tested for weed density) was recorded as disease incidence. Then, the isolation and confirmation of the pathogen was performed in the laboratory (Kakhki et al., 2022). The disease incidence was assessed at one-leaflet seedling, three-leaflet seedling, flowering, podding and maturity stages. In the next step, 10 bean plants, which were assessed for the disease incidence at each of five assessment times, were used to measure bean plant dry matter per plot. The number of pods per plant and seeds per pod were counted for 10 randomly assessed plants per plot at maturity stage.

### Statistical analysis

A total of 256 (128 in 2015 and 128 in 2016) experimental plots were examined for bean dry matter, *Rhizoctonia* root rot, and weed density progress based on the parameters estimated by the best model fitted to the disease, bean and weed development datasets. To improve the degrees of freedom, the datasets for the two study years were pooled. To fit the best regression model, the seasonal measurements of the development of either variable for the two bean cultivars grown under diverse sowing-date and weed-control treatments were examined using the six following standard models: exponential, logistic, Gompertz, linear-by-linear, quadratic-by-linear, and Gaussian (**Table 1**). All the statistical analyses were performed using the GENSTAT (VSN International, Oxford, UK), which fitted standard curves according to maximum likelihood. The best model was fitted considering the following criteria: the co-efficient of determination (R^2^), Fisher’s test, and associations of fitted values with observed values (Brusco and Stahl, 2005; Tabande and Naseri, 2020).

**Table 1:** Standard regression models to characterize development of bean dry matter, *Rhizoctonia* root rot, and weed density in two bean cultivars grown under different planting date and weed control treatments.

Models	Year	Dry matter		*Rhizoctonia* root rot		Weed density	
		*R* ^2^	*F* prob.	*R* ^2^	*F* prob.	*R* ^2^	*F* prob.
Exponential	2015	0.99	< 0.001	0.80	0.198	0.91	< 0.001
	2016	0.96	< 0.001	0.87	0.005	0.74	0.560
Logistic	2015	0.99	nd^a^	0.83	nd	0.82	nd
	2016	0.99	nd	0.86	nd	0.58	nd
Gompertz	2015	0.99	nd	0.88	nd	0.73	nd
	2016	0.99	nd	0.87	nd	0.49	nd
Linear-by-linear	2015	0.99	< 0.001	0.79	0.225	0.98	< 0.001
	2016	0.96	< 0.001	0.88	0.003	0.95	< 0.001
Linear-by-quadratic	2015	0.99	nd	0.99	nd	1.00	nd
	2016	1.00	nd	1.00	nd	1.00	nd
Gaussian	2015	0.99	nd	0.99	nd	0.90	nd
	2016	0.99	nd	0.97	nd	0.94	nd

a nd = Not detected by statistical procedure.

The Kruskal-Wallis test is a nonparametric (distribution free) test, and is used when the assumptions of one-way ANOVA (data normality, homogeneity and independency) are not met. The normality of data variance was determined using kurtosis and skew tests. However, a high heterogeneity in datasets was required to improve predictive values of variables involved in the regression model (Kranz, 2003). Thus, the heterogeneous bean-disease-weed datasets were subjected to the Kruskal-Wallis one-way ANOVA to rank different levels of planting date and weed control treatments. The levels of planting date factor were defined as follows: early level involving two dates of 10-15 May and 26-31 May, and late level involving two dates of 10-15 June and 25-30 June. The weed control practices were classified as follows: herbicide use class applying either Imazethapyr or Trifluralin, hand-weeding class without weeds, and not treated class or control plots. Experimental data for the two bean cultivars, Talash and COS16, were pooled due to slight differences across treated plots. Then, simple correlations between the continuous variables of bean dry matter, *Rhizoctonia* root rot, and weed density descriptors were examined. Loading values indicating the associations of variables with the principal component (with an eigenvalue or proportion of data variance ≥ 1.0), were regarded significant if they were ≥ 0.35 (Kranz, 2003).

Considering the interrelationships among the predictors obtained from the PCA (Tabande and Naseri, 2020), a descriptive regression of bean production was modeled. Principal components with significant contributions into the bean-*Rhizoctonia*-weed interaction were involved in the regression model. Stepwise process used the two criteria of adjusted coefficient of determination (R^2^) and Mallows Cp to fit the best predictors (Brusco and Stahl, 2005). Further to minimizing collinearity among variables by using principal components as predictors of a regression model (Tabande and Naseri, 2020), different principal components were considered independent. Therefore, such bean-*Rhizoctonia*-weed predictors and their two-way interactions were selected to be used in the multivariate regression. Then, the graphical appraisal of normally distributed residuals, F-test and R^2^ were checked for the best model fitness (Tabande and Naseri, 2020).

## Results

### Model fitting

Due to the lack of significant effect of study year, bean-disease-weed datasets for the two years of current study were pooled. This study examined six types of standard regressions to model the development of bean dry matter, *Rhizoctonia* root rot, and weed density across 256 experimental plots during the two growing seasons (**Table 1**). Among the standards models studied, an exponential model or asymptotic regression curve provided the best fit to bean-dry-matter data collected from bean cultivars grown under different planting date and weed control treatments over the two growing seasons. For the exponential model (*a* + b*(r**x)), *a* refers to the constant term, *b* to the bean-dry-matter increase factor, *r* to the rate of bean-dry-matter increase, and *x* is the time intervals (day) between assessments. This function represents a slow increase of bean dry matter at first, followed by a more rapid increase without bound when *r* is greater than 1.

The linear-by-linear regression model (*a* + *b*/(1 + d*x)) was fitted to *Rhizoctonia* root rot incidence data as the best curve. This model with a bimodal distribution described *a* as the constant term, *b* as the disease increase factor, *d* as the strength of regression model (disease progress rate), and *x* as the time (day) between measurements. The linear-by-linear model represents a rapid initial increase/decline of *Rhizoctonia* root rot incidence, and then a subsequently slow decline/recovery to an approximate equilibrium.

The linear-by-linear model (*a* + *b*/(1 + d*x)) was also fitted to weed density data as the best weed development curve. In this model, *a* is the constant term, *b* is the weed density increase factor, *d* is the strength of regression (density progress rate), and *x* is the time intervals. This regression curve indicates a rapid initial increase/decline of weed density followed by a slow decline/recovery.

The exponential (*a*, *b* and *r*) and linear-by-linear (*a*, *b* and *d*) parameters were estimated for each field plot based on the bean-disease-weed datasets. These model fitted parameters were considered as predictors to estimate bean dry matter, *Rhizoctonia* root rot, and weed density development over the growing season for each field plot. The average, standard deviation, minimum and maximum values were provided for the three exponential parameters of bean dry matter and three linear-by-linear parameters of disease incidence and weed density estimators in two cultivars of bean planted at different dates (**Table 2**). Greater standard deviations than the average values obtained for these variables showed diverse ranges of data variations.

**Table 2:** Average, standard deviation, and range values obtained for metric variables of bean dry matter, *Rhizoctonia* root rot incidence, and weed density development in bean cultivars grown under different planting dates and weed control treatments.

Descriptors		Average	Standard deviation	Range
Bean dry matter	Exponential parameter *a*	-139	628	-3845.00 to 449.00
	Exponential parameter *b*	125	617	-474.00 to 3765.00
	Exponential parameter *r*	4.4	7.91	0.69 to 39.40
*Rhizoctonia* root rot	Linear-by-Linear parameter *a*	30	190.7	-354.00 to 1033.00
	Linear-by-Linear parameter *b*	-31.3	201.5	-1063.00 to 334.00
	Linear-by-Linear parameter *d*	-0.254	1.26	-7.00 to 3.50
Weed density	Linear-by-Linear parameter *a*	60.8	73.3	0.00 to 310.00
	Linear-by-Linear parameter *b*	-50.3	310.2	-2395.00 to 219.00
	Linear-by-Linear parameter *d*	-0.28	3.22	-8.00 to 23.00

### Kruscal-Wallis one-way ANOVA

According to the results of Kurtosis and skew tests, the distributions of bean dry matter, disease incidence and weed density datasets were normal. According to the Kruscal-Wallis one-way ANOVA results, the exponential parameter *r* estimated for the development of bean dry matter was affected (H adjusted = 1.8; *Chi* prob. = 0.053) by planting date and weed control factors (**Table 3**). Experimental plots neither treated with herbicides nor hand-weeded indicated the lowest rankings of bean dry matter increase rate (the exponential parameter *r*) at both of the early and late plantings. Hand-weeding classes defined for both levels of the planting date factor showed greater rankings of dry matter increase rate when compared with the herbicide use classes. The constant term of *Rhizoctonia* root rot development (the linear-by-linear parameter *a*) was also affected by the planting date and weed control factors (H adjusted = 7.5; *Chi* prob. = 0.048). This suggested lower initial disease rankings for either herbicide use or hand-weeding classes than the class of not treated plots (control) planted at early and late dates. Furthermore, late plantings indicated lower initial disease rankings than early plantings at each class of the weed control factor. The planting date and weed control factors affected all the three weed density development descriptors, linear-by-linear parameters *a*, *b* and *d*. The initial weed density or the constant term of the linear-by-linear model of weed density development over the growing seasons had the lowest and highest rankings for the hand-weeding and not treated classes, respectively, at both early and late planting dates. Due to negative estimations of linear-by-linear parameters *b* and *d* by the weed density development model, the lowest and highest rankings were detected for not treated and hand-weeding classes, respectively, at both planting date levels. For pod and seed production, planting date and weed control factors affected pod number per plant (H adjusted = 15.8; *Chi* prob. = 0.007) and seed number per pod (H adjusted = 17.5; *Chi* prob. = 0.004). Lower and higher pod and seed production rankings at each planting date were obtained for not treated and hand-weeding classes of the weed control factor, respectively. In addition, herbicide use and hand-weeding classes indicated a greater pod and seed productivity in early plantings than late plantings (**Table 3**). Whereas, the class of not treated plots showed higher productivity rankings in late planted beans in comparison with early planted beans.

**Table 3:** Analysis of development of bean dry matter, *Rhizoctonia* root rot incidence, and weed density using Kruskal-Wallis one-way ANOVA for bean cultivars grown under different planting date and weed control treatments.

Planting date levels	Weed control treatments	Bean dry matter			*Rhizoctonia* root rot			Weed density			Yield	
		Exp.a	Exp.b	Exp.r	Ll.a	Ll.b	Ll.d	Ll.a	Ll.b	Ll.d	Pod	Seed	
Early	Herbicide use	30.3	34.8	34.0	35.8	35.1	30.1	38.3	26.1	32.1	39.1	40.7	
	Hand-weeding	29.8	33.3	37.5	38.4	24.9	33.3	8.5	47.5	53.5	45.1	48.7	
	Not treated	35.3	31.3	26.8	39.9	26.8	37.6	53.4	11.0	29.0	16.6	21.8	
Late	Herbicide use	33.4	31.4	31.8	24.4	38.2	34.0	30.4	37.2	20.6	35.9	28.9	
	Hand-weeding	28.3	35.5	34.8	24.6	36.1	28.5	8.5	47.5	53.5	27.8	32.8	
	Not treated	39.5	27.5	29.5	36.7	25.5	32.5	52.3	27.4	18.8	20.5	17.6	
Mean adjusted *H*		2.2	1.1	1.8	7.5	5.3	1.4	48.1	24.9	32.1	15.8	17.5	
Ranking *Chi* prob.		ns	ns	0.053	0.048	ns	ns	0.001	0.001	0.001	0.007	0.004	

Exp, Exponential parameter; Ll, linear-by-linear parameter; ns, Not significant; Pod, pod number per plant; Seed, seed number per pod.

### Correlation analysis

Based on the correlation analysis results, there was a significantly negative relationship (P *≤ 0*.05) between the exponential parameters *a* and *b* estimated for the development of bean dry matter (**Table 4**). For *Rhizoctonia* root rot development over the two growing seasons studied, the linear-by-linear parameter *b* was negatively linked (P *≤ 0*.05) to the exponential parameters *a* and *d*. From the weed density progression variables, only linear-by-linear parameter *b* negatively corresponded (P *≤ 0*.05) with linear-by-linear parameter *d*. The seed number per pod variable was negatively correlated (P *≤ 0*.05) with the linear-by-linear parameter *a* estimated for the weed density progress variable. There was a significantly positive correlation between the pod number per bean plant and the seed number per pod (**Table 4**).

**Table 4:** Correlations between descriptors of plant production, *Rhizoctonia* root rot, and weed development in bean cultivars planted under different dates and weed control treatments.

Descriptors		BDM*a* ^a^	BDM*b*	BDM*r*	RRR*a*	RRR*b*	RRR*d*	WD*a*	WD*b*	WD*d*	PP	SP
Bean dry matter	Exponential parameter *a*	1.00										
(BDM)	Exponential parameter *b*	-0.99^b^	1.00									
	Exponential parameter *r*	0.11	-0.08	1.00								
*Rhizoctonia* root rot	Linear-by-Linear parameter *a*	0.03	-0.03	-0.05	1.00							
(RRR)	Linear-by-Linear parameter *b*	-0.03	0.03	0.05	**-0.97**	1.00						
	Linear-by-Linear parameter *d*	-0.01	0.01	-0.00	0.04	**-0.28**	1.00					
Weed density	Linear-by-Linear parameter *a*	0.19	-0.21	-0.19	0.01	-0.21	0.06	1.00				
(WD)	Linear-by-Linear parameter *b*	-0.05	0.04	0.06	-0.05	0.08	-0.09	-0.10	1.00			
	Linear-by-Linear parameter *d*	0.01	-0.01	-0.16	0.07	-0.11	0.11	0.03	**-0.96**	1.00		
Pod no./Plant (PP)		-0.17	0.16	-0.08	-0.05	0.05	0.07	-0.17	-0.08	0.11	1.00	
Seed no./Pod (SP)		0.07	-0.06	-0.03	0.09	-0.09	0.03	**-0.26**	0.08	-0.02	**0.31**	1.00

a Italic letters following abbreviations refer to model parameters.

b Bold numbers refer to significance at 0.05 probability level.

### Principal component analysis

The PCA using a correlation matrix indicated associations of relevant bean-disease-weed development, pod-seed production indicators with principal components estimating linear combinations of the variables. From the PCA, the five principal components accounted for 79.5% of the variation in bean dry matter, *Rhizoctonia* root rot, and weed density development data obtained from two commercial bean cultivars planted at different planting dates and weed control treatments during the two seasons as shown in **Table 5**. The first principal component justified 20.5% of the total data variance. This factor provided the positively moderate loadings for the correspondence of the exponential parameter *a* of bean dry matter, linear-by-linear parameter *a* and *d* estimated for *Rhizoctonia* root rot and weed density, respectively. The linear-by-linear parameter *b* estimated for either *Rhizoctonia* root rot and weed density were negatively associated with the first principal component. Thus, this factor of PCA test determined the indirect association of linear-by-linear parameter *b* with the linear-by-linear parameters *a* and *d* significantly contributed. This suggested the association of a lower linear-by-linear parameter *b* (more effective *Rhizoctonia* root rot or weed increase factor) with greater linear-by-linear parameter *a* (initially more intense *Rhizoctonia* root rot) and *d* (weed density progress rate).

**Table 5:** Principal component analysis of plant production, *Rhizoctonia* root rot, and weed development in bean cultivars planted at different dates and weed control treatments.

Variables		Principal components				
		1	2	3	4	5
Bean dry matter	Exponential parameter *a*	0.35^a^	**-0.55**	-0.05	0.22	-0.03
	Exponential parameter *b*	-0.34	**0.55**	0.05	-0.20	0.06
	Exponential parameter *r*	-0.09	-0.16	0.08	0.15	**0.78**
Rhizoctonia root rot	Linear-by-Linear parameter *a*	**0.42**	0.19	**0.50**	-0.09	0.06
	Linear-by-Linear parameter *b*	**-0.45**	-0.21	**-0.49**	0.09	-0.04
	Linear-by-Linear parameter *d*	0.18	0.13	0.07	0.03	-0.10
Weed density	Linear-by-Linear parameter *a*	0.18	-0.18	-0.13	**-0.45**	**-0.44**
	Linear-by-Linear parameter *b*	**-0.40**	-0.26	**0.49**	-0.03	-0.18
	Linear-by-Linear parameter *d*	**0.40**	0.31	**-0.47**	0.07	0.12
Pod no./Plant		-0.07	0.26	-0.06	**0.50**	-0.31
Seed no./Pod		0.02	0.07	0.17	**0.65**	-0.22
Eigenvalues		2.3	2.1	1.9	1.4	1.1
Variation (%)		20.5	19.2	16.9	13.0	9.9
Accumulated variation (%)		20.5	39.7	56.6	69.6	79.5

a A bold number indicates a significant loading value ≥ 0.35.

The second principal component, accounting for 19.2% of the total bean-disease-weed data variance, demonstrated the moderately significant contributions of the initial bean dry matter (constant term or exponential parameter *a*) and the dry matter increase factor (exponential parameter *b*) to characterize a diverse range of bean-disease-weed development curves developed during two growth seasons (**Table 5**). This factor detected the association of a lower parameter *b* of exponential (less effective bean dry matter increase factor) models with a higher exponential parameter *a* (greater initial bean dry matter).

The third principal component justified 16.9% of the total bean-disease-weed data variance (**Table 5**). This factor provided the significantly positive contributions of the initial *Rhizoctonia* root rot (linear-by-linear parameter *a*) and weed density increase factor (linear-by-linear parameter *b*), and negative contributions of the disease increase factor (linear-by-linear parameter *b*) and weed density increase rate (linear-by-linear parameter *d*). This suggested the indirect association of parameter *b* (*Rhizoctonia* root rot or weed density increase factor) with the parameters *a* (the initial disease) and *d* (weed density increase rate) of linear-by-linear models.

The forth principal component justified 13.0% of the total dataset variance, detecting significant associations of the two bean productivity variables, pod number per plant and seed number per pod, and the initial weed density (linear-by-linear parameter *a*; **Table 5**). This demonstrated the correspondence of the initially less dense weed populations with greater pod numbers per plant and seed numbers per pod produced by the two bean cultivars studied. The fifth principal factor explained 9.9% of the data variance and received a highly significant contribution from the exponential parameter *r* (the bean dry matter increase rate) and a moderately significant contribution from linear-by-linear parameter *a* estimated for weed density. This suggested the correspondence of the initially less dense weed populations with a greater bean dry matter increase rate (exponential parameter *a*; **Table 5**). This PCA identified not only the significant indicators estimating the progression of bean dry matter, *Rhizoctonia* root rot and weed density, but also the joint associations between the crop production, disease and weed development.

### Multivariate regression analysis

The multivariate regression analyses (F probability = 0.001; R^2^ = 0.49) demonstrated that 49% of variations in pod and seed production in bean cultivars treated with different planting dates and weed control methods across 256 experimental plots were explained according to the bean dry matter, *Rhizoctonia* root rot and weed density development descriptors (**Table 6**). Singular and two-way interactions of bean dry matter exponential parameter *a*, *b* and *r*, and linear-by-linear parameters *a* and *b* estimated for *Rhizoctonia* root rot and weed density development corresponded significantly with the number of pod/plant and seed/pod in the bean crops studied during the two growing seasons. There was no significant difference between the observed and fitted values of pod and seed regression models developed based on the bean growth, disease and weed parameters. Therefore, bean pod and seed production was estimated using the predictors described for over-season development of bean growth, disease and weed examined at field-plot scale (**Table 6**).

**Table 6:** Multivariate regression analysis of pod and seed production based on principal component analysis of bean growth, *Rhizoctonia* root rot, and weed descriptors in two cultivars sown under different dates and weed control treatments.

Variables	Model	Parameter estimate	Standard error	*t*-probability
Bean dry matter EXP*b* × *Rhizoctonia* root rot LL*a*	Pod no./Plant	-0.00007	0.00009	0.431
	Seed no./Pod	-0.00004	0.00004	0.340
*Rhizoctonia* root rot LL*a* × *Rhizoctonia* root rot LL*b*	Pod no./Plant	-0.00001	0.00001	0.123
	Seed no./Pod	-0.00001	0.00001	0.100
*Rhizoctonia* root rot LL*b* × Weed density LL*b*	Pod no./Plant	0.00030	0.00025	0.246
	Seed no./Pod	0.00011	0.00010	0.288
Bean dry matter EXP*a* × Bean dry matter EXP*b*	Pod no./Plant	-0.00001	0.00000	0.024
	Seed no./Pod	-0.00001	0.00000	0.046
*Rhizoctonia* root rot LL*a* × Weed density LL*b*	Pod no./Plant	0.00042	0.00036	0.257
	Seed no./Pod	0.00015	0.00014	0.288
Weed density LL*a*	Pod no./Plant	0.04400	0.01170	<0.001
	Seed no./Pod	0.01747	0.00465	<0.001
Bean dry matter EXP*r* × Weed density LL*a*	Pod no./Plant	0.00895	0.00396	0.028
	Seed no./Pod	0.00378	0.00158	0.020

a EXPa, Exponential parameter a; EXPb, exponential parameter b; EXPr, exponential parameter r; LLa, Linear-by-Linear parameter a; LLb, Linear-by-Linear parameter b.

## Discussion

Sustainable bean production strictly requires the development of an integrated environmental friendly crop management program (Naseri, 2019a). To meet this requirement, it was attempted in the current research to study over-season fluctuations in plant growth, *Rhizoctonia* root and stem rot, and weed density in two bean cultivars (with different habits) treated with different planting dates and weed control practices across experimental field plots. Hence, we firstly needed to determine the best indicators of plant dry matter, *Rhizoctonia* root rot, and weed density development in bean cultivars treated with diverse planting dates and weed control methods. This experimental design provided a diverse range of variations in the bean-disease-weed progression over time due to treating field plots with different planting dates and weed control practices. Such a noticeable variability in data helps to increase the predictive value of a disease progress curve element (Kranz, 2003; Naseri and Veisi, 2019). The current findings suggested exponential and linear-by-linear parameters as the best predictors of plant dry matter, *Rhizoctonia* root rot, and weed density progress in early to very late-planted bean cultivars under gone different weed control practices studied during the two growing seasons. With the best of our knowledge, the current field-scale findings demonstrated the notable predictive values of bean-disease-weed development variables to improve the accuracy of future estimation of bean production, *Rhizoctonia* root rot, and weed to develop more effective crop management programs and higher yield levels.

In China, Tan et al. (2007) developed Gompertz and logistic models to simulate rice sheath blight progress dynamics according to disease severity ratings. Campbell et al. (1980) described the final disease severity and first-difference regression linear coefficient as predictors of bean root rot progress curves to improve accuracy of epidemiological findings. In Nigeria, Chikoye et al. (2006) estimated the efficiency of a new formulation of Atrazine herbicide to manage weed populations in maize fields using an exponential model. However, none of previous studies simulated the progression of bean growth, *Rhizoctonia* root rot, and weed according to the best indicators fitted to field-scale datasets. Therefore, the present findings on interrelationships among the crop productivity, *Rhizoctonia* root and stem rot, and weed development using the three exponential and three linear-by-linear parameters as the best indicators of bean dry matter, disease, and weed dynamics in bean cultivars appears to be the first report. Such information must add a further value to future estimations of bean production in farming systems infested by root rots and weeds.

According to the Kruskal-Wallis one-way ANOVA results, the use of herbicides in early planted plots restricted the development of weed density during the growing season by 28%. Whereas, late planting improved the weed control efficiency of herbicides by reducing weed density by 42% based on the linear-by-linear parameter *a* estimates for herbicide use and not treated classes of weed control factor. For the over-season progression of *Rhizoctonia* root rot, late plantings of bean cultivars decreased the disease development by 8-36% according to the linear-by-linear parameter *a* estimates for the three weed control treatments. Although the dry matter, pod and seed production of bean cultivars for the herbicide use and hand-weeding treatments were greater than not treated control plots, late planting improved bean dry matter and pod production in plots neither treated with herbicides nor hand-weeded by 10-24%. To the best of our knowledge, such new information on significant effects of planting date on the current bean dry matter and productivity, *Rhizoctonia* root rot and weed density datasets collected from experimental field plots treated with various planting dates and weed control practices may emphasize on the necessity of proper planting date to be considered as an sustainable disease and weed management method into future bean cultivation programs.

Associations of bean production with agronomic practices and soil conditions (Naseri and Veisi, 2019), bean growth (Manschadi et al., 1998), *Rhizoctonia* root rot (Campbell et al., 1980), weed populations (Ghamari and Ahmadvand, 2012) had been reported earlier. The treatments of Clethodim herbicide and *Trichoderma* spp. reduced weeds and *Rhizoctonia* root rot and improved plant growth and yield parameters in two faba bean (*Vicia faba* L.) cultivars grown in naturally infested field soils (El-Dabaa et al., 2019). However, none of the previous studies reported the best indicators of bean growth, disease and weed dynamics over the growing season in association with the pod and seed production. As far as we know, the associations of pod and seed production in beans with the best indicators of plant dry matter, *Rhizoctonia* root rot, and weed density development at the scale of an experimental field is reported for the first time. This multivariate analysis demonstrated that the independent principal components explained 80% of total variance in bean-disease-weed datasets obtained during the two growing seasons at plot scale.

It could be concluded that a main (80%) part of variations in the crop growth and production, disease and weed development in bean cultivars grown under different planting dates and weed control treatments was explained according to the eleven bean-disease-weed descriptors in the current study. In addition to the above-mentioned findings, the present study determined the similar descriptive values for bean dry matter, *Rhizoctonia* root rot, and weed density indicators. The PCA results demonstrated the significant contribution of bean dry matter, disease and weed development into the first principal component explaining 21% of total data variance, suggesting the noticeable interrelationships among these bean-disease-weed variables. Furthermore, the multivariate regression models developed for pod and seed production explained a considerable part (49%) of variations in the bean-disease-weed datasets. This suggested that those model parameters estimated in the current research were responsible for about half of the variability in bean growth, *Rhizoctonia* root rot and weed density during the two growing season. Such information may assists with more accurately monitoring bean-disease-weed development and predicting bean productivity for sustainable crop management programs in future.

## Data availability statement

The raw data supporting the conclusions of this article will be made available by the authors, without undue reservation.

## Author contributions

SN: Designing and performing the work, and BN: interpreting the data and writing the paper. All authors contributed to the article and approved the submitted version.

## Funding

This study was funded by the Iranian Agricultural Research, Education & Extension Organization, projects 4-47-16-88113 and 4-47-16-90070.

## Acknowledgments

Authors appreciate the technical assistance in the field.

## Conflict of interest

The authors declare that the research was conducted in the absence of any commercial or financial relationships that could be construed as a potential conflict of interest.

## Publisher’s note

All claims expressed in this article are solely those of the authors and do not necessarily represent those of their affiliated organizations, or those of the publisher, the editors and the reviewers. Any product that may be evaluated in this article, or claim that may be made by its manufacturer, is not guaranteed or endorsed by the publisher.
